# Supplementary material for: Sam68 promotes self-renewal and glycolytic metabolism in mouse neural progenitor cells by modulating Aldh1a3 pre-mRNA 3'-end processing
Source: eLife. 2016 Nov 15;5:e20750. doi: 10.7554/eLife.20750 (PMC5122457; doi:10.7554/eLife.20750)
Supplement: Supplementary file 1. — DOI: http://dx.doi.org/10.7554/eLife.20750.022 [file elife-20750-supp1.docx]

**Supplementary File 1.** List of the oligonucleotides used as PCR primers and siRNAs in the study.

| OLIGONUCLEOTDE NAME | SEQUENCE 5’--> 3’ | EXPERIMENT |
| --- | --- | --- |
| PCR_SAM68 Fw | tggaccacctagaggagctt | RT-PCR |
| PCR_SAM68 Rv | cagaagccagaatgcagagtt | RT-PCR |
| SOX2 Fw | cacaactcggagatcagcaa | RT-PCR |
| SOX2 Rv | ctccgggaagcgtgtactta | RT-PCR |
| TUJ-1 Fw | gcggcaactatgtaggggac | RT-PCR |
| TUJ-1 Rv | gcctgaataggtgtccaaaggc | RT-PCR |
| GAPDH Fw | aactttggcattgtggaagg | RT-PCR/ qPCR |
| GAPDH Rv | cacattgggggtaggacac | RT-PCR/ qPCR |
| L34 Fw | ggtgctcagaggcactcaggatg | RT-PCR/ qPCR |
| L34 Rv | gtgctttcccaaccttcttggtgt | RT-PCR/ qPCR |
| ANGPT2 Fw | agtagcatcagccaaccagg | ARRAY VALIDATION |
| ANGPT2 Rv | gaccacatgcgtcaaaccac | ARRAY VALIDATION |
| GFAP Fw | tttgcagacctcacagacgc | ARRAY VALIDATION |
| GFAP Rv | ttggcggcgatagtcgttag | ARRAY VALIDATION |
| EN2 Fw | aatcaagaaagccacgggca | ARRAY VALIDATION |
| EN2 Rv | ctactcgctgtccgacttgc | ARRAY VALIDATION |
| LHX Fw | ctaccccagcagccaaaaga | ARRAY VALIDATION |
| LHX Rv | ttggcatcgggattgtggtt | ARRAY VALIDATION |
| MBP Fw | ctcacacacgagaactaccca | ARRAY VALIDATION |
| MBP Rv | gttttcatcttgggtccggc | ARRAY VALIDATION |
| CLDN11 Fw | catggccactggtctctacc | ARRAY VALIDATION |
| CLDN11 Rv | agaacggaggcagcaatcat | ARRAY VALIDATION |
| LSLR Fw | gcagactgtgcctatcgtga | ARRAY VALIDATION |
| LSLR Rv | ccgagcggatctcattgtgt | ARRAY VALIDATION |
| PLP1 Fw | ccacctgtttattgctgcgtt | ARRAY VALIDATION |
| PLP1 Rv | gacggcgaagttgtaagtgg | ARRAY VALIDATION |
| UCP2 Fw | ggtcggagataccagagcac | ARRAY VALIDATION |
| UCP2 Rv | ggcattacgggcaacattgg | ARRAY VALIDATION |
| MOBP Fw | ggaccgcaagtacagcatctgc | ARRAY VALIDATION |
| MOBP Rv | cttcttccttggggttgacctgc | ARRAY VALIDATION |
| CNTNAP2 Fw | gctctcgctctggattctca | ARRAY VALIDATION |
| CNTNAP2 Rv | aggacacatggggaagacca | ARRAY VALIDATION |
| S100A1 Fw | caagaaagaactgaaagacctgct | ARRAY VALIDATION |
| S100A1 Rv | tctccgttttcatccagttcct | ARRAY VALIDATION |
| RASAL2 Fw | gctttagctgtagctctgcct | ARRAY VALIDATION |
| RASAL2 Rv | cggagaacattttcagctcgtc | ARRAY VALIDATION |
| NFASC Fw | gctggtatgccgagccaat | ARRAY VALIDATION |
| NFASC Rv | gctacctcacggttgggatt | ARRAY VALIDATION |
| OTX2 Fw | gggctgagtctgaccacttc | ARRAY VALIDATION |
| OTX2 Rv | acagagcttccagaacgtcg | ARRAY VALIDATION |
| SIX3 Fw | ttgcttcaaggagcggactc | ARRAY VALIDATION |
| SIX3 Rv | agttgcctacttgtgtgggg | ARRAY VALIDATION |
| SOX10 Fw | aaccaccccaaagacagagc | ARRAY VALIDATION |
| SOX10 Rv | ttgggtggcaggtattggtc | ARRAY VALIDATION |
| EGFR Fw | cgttccctcaaggagatcag | ARRAY VALIDATION |
| EGFR Rv | gggtgtcccgaagagttttt | ARRAY VALIDATION |
| ALDH1A3 Ex6 Fw | ggaacttccccctgctgatg | RT-PCR |
| ALDH1A3 Ex7 Rv | ctccgggtgtggtgaacatt | RT-PCR |
| ALDH1A3 In7 Rv | ccaccgacgtttcc ttgaga | RT-PCR |
| ALDH1A3 Ex9 Fw | gagtgtggagttcgccaaga | RT-PCR |
| ALDH1A3 Ex10 Rv | ggcaatcctcatgttgtccg | RT-PCR |
| ALDH1A3 Ex2 Fw | tcaacaacgactggcacgaa | qPCR |
| ALDH1A3 Ex3 Rv | ccttgtccacatcgggcttat | qPCR |
| ALDH1A3 Ex10 Fw | aaacccacggtcttctcagat | qPCR |
| ALDH1A3 Ex11 Rv | atcacctcctccaggtttttga | qPCR |
| ALDH1A3 Ex7 Fw | ctcccatccgcagatcaaca | qPCR |
| ALDH1A3 In7Bis Rv | tctcaaggaaacgtcggtgg | qPCR |
| ALDH1A3 Ex7 RACEFw | ctccgggtgtggtgaacatt | 3’ RACE |
| ALDH1A3 Ex7/in7 RACE Fw | accggctccacagaggtacg | 3’ RACE |
| ALDH1A3 In7 alt pol1 Fw | ggcttccaaactctattagcta | RT-PCR |
| ALDH1A3 In7 alt pol2 Fw | agctacatgctgtacaactggt | RT-PCR |
| ALDH1A3 In7 alt pol3 Fw | tgtccacattgcctggacct | RT-PCR |
| GFP-ALDH1A3 FL/Δ Fw | agaagcttatggctaccaccaacggg | ALDH1A3 CLONING |
| GFP-ALDH1A3 FL Rv | agctgcagtcaggggttcttcttctc | ALDH1A3 CLONING |
| GFP-ALDH1A3Δ Rv | agctgcagttagaaaaagagaagcaaatagtattt | ALDH1A3 CLONING |
| pCI- ALDH1A3 FL/Δ Fw | aggaattcatggctaccaccaacggg | ALDH1A3 CLONING |
| pCI- ALDH1A3 FL Rv | aggcggccgctcaggggttcttctcctc | ALDH1A3 CLONING |
| pCI- ALDH1A3Δ Rv | aggcggccgcttagaaaaagagaagcaaatagtattt | ALDH1A3 CLONING |
| siALDH1A3 #1 | ccaggguguucguggaagauu | KNOCKDOWN |
| siALDH1A3 #2 | uggcagagaacuaggagaauu | KNOCKDOWN |
| siALDH1A3 #3 | ccaucaaacucgaggagaauu | KNOCKDOWN |
| 3’RACE RT | ctgatctagaggtaccggatccttttttttttttttttttt | 3’ RACE |
| 3’RACE PCR | ctgatctagaggtaccggatcc | 3’ RACE |
| ALDH1A3 E3 Fw | catcagctggctgaccttgt | CLIP assay |
| ALDH1A3 I3 Rv | gcgaggacagttttccccata | CLIP assay |
| ALDH1A3 E4 Fw | tattttgccgggtgggcaga | CLIP assay |
| ALDH1A3 I4 Rv: | gacagggcccccagtagac | CLIP assay |
| ALDH1A3 I7 1 Fw | acaacatcattaaaaccaagcga | CLIP assay |
| ALDH1A3 I7 1 Rv | acatttggagagctacatgctg | CLIP assay |
| ALDH1A3 I7 2 Fw | atcactgaggcaggtgacca | CLIP assay |
| ALDH1A3 I7 2 Rv | agggcaacaattaggctctgt | CLIP assay |
| ALDH1A3 E11 Fw | tggacaaagcactgaagctgg | CLIP assay |
| ALDH1A3 I11 Rv | tggttgctcagacaacacaaa | CLIP assay |
| ALDH1A3 E13 Fw | ggtttacccctgtggtatggt | CLIP assay |
| ALDH1A3 E13 Rv | tgctgaacacacaatctgaac | CLIP assay |
| ALDH1A3 ex3 Fw | aaagggaccgagcgatcctg | DRB experiment |
| ALDH1A3 in3 Rv | caaacagacccatagccccaca | DRB experiment |
| ALDH1A3 ex7 Fw | ttcctcccatccgcagatcaac | DRB experiment |
| ALDH1A3 in7 Rv | cgtcggtgggtctcaaataatgg | DRB experiment |
| ALDH1A3 in8 Fw | cacacgctcaccacatctaa | DRB experiment |
| ALDH1A3 ex8 Rv | agtcagcatctgcacacacga | DRB experiment |
